# Personal

**Published:** 1910-04

**Authors:** 


### PERSONAL.

---

Dr. Lee Gunn of Hamburg, has been appointed assistant surgeon of the Emergency Hospital of the Sisters of Charity, Pine street, Buffalo.

---

Dr. Thomas Bagley of Buffalo, who has made a trip recently to Philadelphia, Washington, Atlantic City and Old Point Comfort has returned and resumed his professional work.

---

Dr. Edgar J. Gilray, for many years Medical Superintendent of the Erie County Hospital, Buffalo, has removed to Innisfail, Alberta, Canada.

---

Dr. H. Sheridan Baketel, of New York, who for several years has been the advertising manager for the Denver Chemical Co., has resigned that position and has become Vice-president and General Manager of the Thermo Chemical Company.

---

Dr. F. Park Lewis, of Buffalo, will spend the coming summer abroad. He will be accompanied by Mrs. Lewis and their two daughters. The family will occupy their lakeside summer home for a month or two before sailing for Europe.

---

Dr. Henry P. Frost, first assistant physician at the Buffalo State Hospital has been appointed superintendent of the Boston State Hospital for the Insane. This hospital recently passed from the control of the city to the state, and Dr. Frost will be its first permanent head. Before coming to Buffalo he had been connected with the Willard State Hospital. Dr. Frost is a member of the American Medical Association, and the American Medical Psychological Association, as well as the Medical Society of the State, of the County, and of the Buffalo Academy of Medicine. He will take up the active duties of the new place April 15. 1910.

